# Expression of Membrane Progesterone Receptors in Eutopic and Ectopic Endometrium of Women with Endometriosis

**DOI:** 10.1155/2020/2196024

**Published:** 2020-07-13

**Authors:** Edgar Ricardo Vázquez-Martínez, Claudia Bello-Alvarez, Ana Lorena Hermenegildo-Molina, Mario Solís-Paredes, Sandra Parra-Hernández, Oliver Cruz-Orozco, J. Roberto Silvestri-Tomassoni, Luis F. Escobar-Ponce, Luis A. Hernández-López, Christian Reyes-Mayoral, Andrea Olguín-Ortega, Brenda Sánchez-Ramírez, Mauricio Osorio-Caballero, Elizabeth García-Gómez, Guadalupe Estrada-Gutierrez, Marco Cerbón, Ignacio Camacho-Arroyo

**Affiliations:** ^1^Unidad de Investigación en Reproducción Humana, Instituto Nacional de Perinatología-Facultad de Química, Universidad Nacional Autónoma de México, Ciudad de México 11000, Mexico; ^2^Departamento de Genética y Genómica Humana, Instituto Nacional de Perinatología, Mexico; ^3^Departamento de Inmunobioquímica, Instituto Nacional de Perinatología, Mexico; ^4^Subdirección de Reproducción Humana, Instituto Nacional de Perinatología, Mexico; ^5^Departamento de Ginecología, Instituto Nacional de Perinatología, Mexico; ^6^Coordinación de Ginecología Laparoscópica, Instituto Nacional de Perinatología, Mexico; ^7^Departamento de Salud Sexual y Reproducción Humana, Instituto Nacional de Perinatología, Mexico; ^8^Unidad de Investigación en Reproducción Humana, Consejo Nacional de Ciencia y Tecnología (CONACyT)-Instituto Nacional de Perinatología, Ciudad de México 11000, Mexico; ^9^Dirección de Investigación, Instituto Nacional de Perinatología, Ciudad de México 11000, Mexico

## Abstract

Endometriosis is one of the most frequent gynecological diseases in reproductive age women, but its etiology is not completely understood. Endometriosis is characterized by progesterone resistance, which has been explained in part by a decrease in the expression of the intracellular progesterone receptor in the ectopic endometrium. Progesterone action is also mediated by nongenomic mechanisms via membrane progesterone receptors (mPRs) that belong to the class II members of the progesterone and adipoQ receptor (PAQR) family. The aim of the present study was to evaluate the expression at mRNA and protein levels of mPR members in the eutopic and ectopic endometrium of women with endometriosis. Total RNA and total protein were isolated from control endometrium (17 samples), eutopic endometrium (17 samples), and ectopic endometrium (9 samples). The expression of *PAQR7* (mPR*α*), *PAQR8* (mPR*β*), and *PAQR6* (mPR*δ*) at mRNA and protein levels was evaluated by RT-qPCR and Western blot, whereas *PAQR5* (mPR*γ*) gene expression was evaluated by RT-qPCR. Statistical analysis between comparable groups was performed using one-way ANOVA followed by Tukey's multiple comparisons test with a confidence interval of 95 %. The analysis of gene expression showed that *PAQR7* and *PAQR5* expression was lower in both eutopic and ectopic endometrium as compared to the endometrium of women without endometriosis, whereas the expression of *PAQR8* and *PAQR6* was only reduced in eutopic endometrium. Furthermore, mPR*α* and mPR*β* protein content was decreased in the ectopic endometrium of women with endometriosis. Our results demonstrate a decrease in the expression and protein content of mPRs in eutopic and ectopic endometrium of patients with endometriosis, which could contribute to the progesterone resistance observed in patients with this disease.

## 1. Introduction

Endometriosis is defined as the presence of endometrial glands and stroma outside the uterus, which are commonly found in the peritoneal cavity and ovaries [1–3]. Endometriosis is the leading cause of chronic and cyclic pelvic pain in reproductive age women, affecting 10-15 % of women worldwide; pain symptoms include dysmenorrhea, dyspareunia, dysuria, and dyschezia [4, 5]. Infertility is commonly associated with this disease mainly due to physical and molecular disruption in the uterus which in turn reduces implantation capacity and finally increases the risk of pregnancy loss [6]. Moreover, endometriosis negatively impacts women's quality of life by deteriorating their physical, mental, and social wellbeing [7]. The gold standard for the diagnosis of endometriosis is made by laparoscopic inspection with histologic confirmation after biopsy [8]. The aim of endometriosis treatment is to mitigate the symptoms associated with the disease and includes pharmacological therapy with nonsteroidal anti-inflammatory drugs, progestins, oral contraceptives, and gonadotropin-releasing hormone agonists, as well as surgical removal of endometrial implants and the affected tissue; however, endometriosis recurs in at least 5-15 % of the cases after most invasive surgeries [8, 9]. The etiology of this disease is far from being elucidated; however, altered estrogen signaling and progesterone resistance have been identified as the most common hallmarks of this disease [10].

Progesterone resistance in endometriosis has been attributed in part to a decrease in the expression of the B isoform of its intracellular receptor (PR-B) in the endometriotic lesions (ectopic endometrium) of women with the disease [11]. Furthermore, it has been proposed that progesterone resistance leads to an altered eutopic endometrium function in women with endometriosis, which in turn has been associated with pregnancy loss [6]. There is controversy about the alteration in the expression of PR in eutopic endometrium, suggesting that other mechanisms should be involved in progesterone resistance in this tissue [12].

Progesterone induces the decidualization of the endometrium, which is essential for embryo implantation and maintenance of pregnancy [13]. It has been demonstrated that progesterone exerts its actions by activating genomic and nongenomic mechanisms [14, 15]. Genomic action mechanisms are mediated by the PR, which acts as a ligand-dependent transcription factor that regulates the expression of progesterone-responsive genes [16–18]. Moreover, nongenomic action mechanisms are mediated in part by specific receptors localized in the plasma membrane that are not related to PR and are divided into two major groups: the membrane progesterone receptors (mPRs) that belong to the class II members of the progesterone and adipoQ receptor (PAQR) family and the progesterone receptor membrane components (PGRMCs) [19].

mPRs are G protein-coupled receptors that are encoded by five different genes: *PAQR7* (mPR*α*), *PAQR8* (mPR*β*), *PAQR5* (mPR*γ*), *PAQR6* (mPR*δ*), and *PAQR9* (mPR*ε*) [19, 20]. The activation of mPRs is necessary to achieve full effects of progesterone in some responsive tissues or cells to this hormone in which those effects are only partially explained by PR activation [21–23]. Importantly, we and others have demonstrated that the content and activity of these receptors are altered in many diseases, including cancer [24–27]. The expression pattern of mPRs is tissue-specific and their activation by progesterone or by the mPR specific agonist 10-ethenyl-19-norprogesterone (Org OD 02-0) regulates signaling pathways involved in mammary gland development, sexual behavior, ovulation, maintenance of pregnancy, and other processes [19, 21, 28–32]. mPRs are expressed in female reproductive and embryonic tissues, mainly in the endometrium, myometrium, ovaries, and placenta [19, 30, 33, 34]. Particularly, it has been demonstrated that *PAQR7*, *PAQR8*, *PARQ5*, and *PAQR9* are expressed in the endometrium. *PAQR7* expression is induced during the secretory phase of the menstrual cycle, whereas the expression of *PAQR5* and *PAQR9* is decreased during that phase [30]. In addition, *PAQR7* and *PAQR8* expression and the respective protein content are decreased in endometrial cancer compared to adjacent nonaffected endometrium, whereas mPR*γ* protein content is increased in endometrial cancer tissue [35]. To the best of our knowledge, it has not been demonstrated whether gene expression and protein content of mPRs are altered in ectopic lesions and eutopic endometrium of patients with endometriosis.

We hypothesized that the expression of mPRs is decreased in both eutopic and ectopic endometrium of patients with endometriosis compared with the endometrium of women without the disease, similar to that reported in PR. Therefore, the aim of the present study was to evaluate the mRNA expression and protein content of mPRs in eutopic and ectopic endometrium of women with endometriosis and endometrium in control subjects.

## 2. Materials and Methods

### 2.1. Participants and Tissue Collection

Seventeen patients with ovarian endometriosis (confirmed by laparoscopy and histological analysis) and seventeen women without the disease undergoing hysterectomy for benign conditions were recruited. Women included in the present study had regular menstrual cycles and did not take any hormonal treatment (including contraceptives) for at least 3 months before obtaining the sample. This study was approved by the Research and Ethical Committee from the Instituto Nacional de Perinatología in Mexico City, Mexico, reference number IRB00001944 and complied with the 1964 Declaration of Helsinki and its later amendments. All study participants signed informed consent for enrolment in the present study. Samples were collected from November 2016 to October 2019. A total of seven tissue biopsies from ovarian endometrioma, two tissue biopsies from peritoneum lesions, and seventeen biopsies from eutopic endometrium were obtained from women with a diagnosis of ovarian endometriosis at the time of resection surgery. Seventeen endometrial biopsies were obtained from women without endometriosis (controls). Endometrial samples from patients (eutopic) and controls were obtained using a Pipelle suction curette. Almost half of the samples were obtained during the proliferative phase of the menstrual cycle. Once obtained, samples were immediately transferred and conserved in RNA later (Qiagen) at -20 °C until RNA and protein isolation.

### 2.2. RNA Isolation and RT-qPCR

RNA isolation was performed using the RNeasy Fibrous Tissue Kit (74704, Qiagen) following the manufacturer's instructions. RNA Integrity Number (RIN) was determined in an Agilent 2100 Bioanalyzer (Agilent Technologies). All samples included in the present study showed a RIN score > 7.0. RNA was quantified using a NanoDrop 2000 spectrophotometer (Thermo Fisher Scientific). cDNA was obtained from 2 *μ*g of total RNA using the M-MVL reverse transcriptase and oligo-dT_12-18_ primers according to the manufacturer's instructions (28025013, Thermo Fisher Scientific). 20 ng of cDNA was amplified using the StepOnePlus PCR system (Thermo Fisher Scientific) and the Power SYBR Green PCR Master Mix (4367659, Thermo Fisher Scientific) following the manufacturer's protocol.  Table 1 describes the oligonucleotides used in the present study. Negative controls with non-retrotranscribed RNA and without cDNA were included in all the experiments. Relative quantification of gene expression was performed by the *ΔΔ*Ct method (relative to the average *Δ*CT values of the control group), in which *18S* ribosomal RNA was used as the endogenous reference gene. All PCR reactions generated a single product of the expected size, as evidenced by melting curve analysis and agarose gel electrophoresis, respectively.

### 2.3. Protein Isolation and Western Blot

Tissues from biopsies were homogenized with a Polytron homogenizer using a T-PER buffer (FNN0071, Thermo Fisher Scientific) supplemented with a protease inhibitor cocktail (p8340, Sigma-Aldrich). Total proteins were obtained by centrifugation at 22000 g, at 4 °C for 5 min and quantified using a NanoDrop 2000 spectrophotometer (Thermo Scientific).

Protein samples (50 *μ*g) were separated on a 12 % *v*/*v* SDS-PAGE at 80 V then were transferred to PVDF membranes (Millipore) in semidry conditions at room temperature at 25 V for 30 min. Membranes were blocked with 5 % *w*/*v* of bovine serum albumin (BSA) at 37 °C under constant agitation for 2 h. Then, they were incubated with the primary antibodies: goat polyclonal anti-mPR*α* and mPR*β* (Santa Cruz Biotechnology 1 *μ*g/mL; sc-50111 and sc-50109 [C-20]) and rabbit polyclonal anti mPR*δ* (Novus Biologicals 1 *μ*g/mL; NPB1-59428) or mouse monoclonal anti *γ*-tubulin (Santa Cruz Biotechnology 1 *μ*g/mL; sc-5286), at 4 °C for 48 h. Blots were then incubated with a rabbit anti-goat secondary antibody (Santa Cruz Biotechnology 1 : 10000; sc-2768) and goat anti-mouse secondary antibody (Santa Cruz Biotechnology 1 : 10000; sc-2005) conjugated to horseradish peroxidase at room temperature under constant agitation for 45 min.

Chemiluminescence signals were detected, exposing membranes to Kodak BioMax Light Films (Sigma-Aldrich) using the SuperSignal West Femto as peroxidase substrate (Thermo Scientific). The band density for the antigen-antibody complex was calculated as the area under a peak in a semiquantitative way using a 14.1-megapixel digital Canon camera (SD1400IS, Canon) and the ImageJ 1.45S software (National Institutes of Health).

### 2.4. Statistical Analysis

All data were analyzed and plotted using the GraphPad Prism 6.0e program (GraphPad Software, Inc., USA). Statistical analysis between comparable groups was performed using one-way ANOVA followed by Tukey's multiple comparisons test with a confidence interval of 95 %.

## 3. Results

### 3.1. Demographical and Clinical Data

There were no differences in the demographic data and most of the clinical data between patients with endometriosis and women without the disease (Table 2). As expected, the only difference between the study groups relies on the fact that all women with endometriosis manifested pelvic pain, which was not reported by the control women. Almost half of the women with endometriosis included in the present study had severe endometriosis and presented a previous endometriosis surgery. Other lesions were found in most of the patients involved in the present study, which included bilateral endometriomas, adhesions, peritoneal endometriosis, uterosacral ligaments, and endometriotic lesions in the appendix and pelvic wall. It was difficult to determine the precise phase of the menstrual cycle of some women recruited in the present study, since most of them did not provide that information in the medical record.

### 3.2. mPR Coding Genes Are Downregulated in Ectopic and Eutopic Tissue of Women with Endometriosis

**Figure 1:**
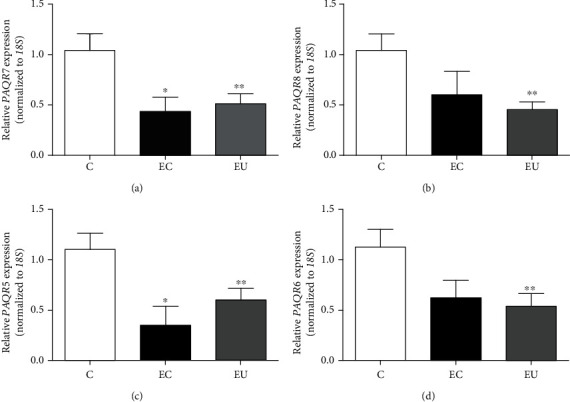
Expression levels of *PAQR* genes in ectopic and eutopic endometrium of patients with endometriosis. Total RNA was extracted from each tissue biopsy, and RT-qPCR was performed to evaluate the relative expression of (a) *PAQR7*, (b) *PAQR8*, (c) *PAQR5*, and (d) *PAQR6* genes, which was calculated by the *ΔΔ*Ct method. Data were normalized using *18S* transcript as a constitutive gene expression control. Results are expressed as mean ± S.E.M. Controls (C, *n* = 17), ectopic (EC, *n* = 9), and eutopic (EU, *n* = 17) endometrium of women with endometriosis. ∗*P* < 0.05 vs C; ∗∗*P* < 0.05 vs C.

Using RT-qPCR, we observed that the expression of *PAQR7*, *PAQR8*, *PAQR5*, and *PAQR6* genes was significantly downregulated in the eutopic endometrium of patients with endometriosis compared with the endometrium of women without the disease. Moreover, the expression of *PAQR7* and *PAQR5* was significantly reduced in ectopic endometrium (Figure 1). Interestingly, very similar expression levels of *PAQR7*, *PAQR8*, *PAQR5*, and *PAQR6* genes were observed between eutopic and ectopic endometrium.

### 3.3. mPR*α* and mPR*β* Content Is Decreased in Ectopic Endometrium of Patients with Endometriosis

**Figure 2:**
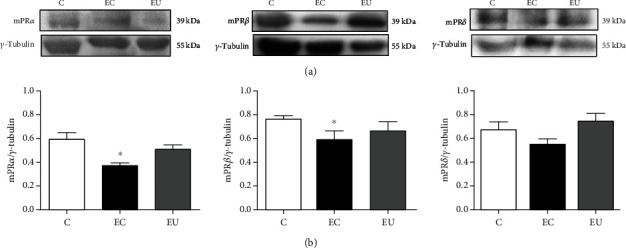
Protein content of mPRs in ectopic and eutopic endometrium of patients with endometriosis. Tissue biopsies were lysed, and proteins (50 *μ*g) were separated by electrophoresis on 12% SDS-PAGE. Gels were transferred to PVDF membranes and then incubated with antibodies against mPR*α*, mPR*β*, mPR*δ*, or *γ*-tubulin (used for normalization). (a) Representative images and (b) densitometric analysis of mPR*α*, mPR*β*, or mPR*δ* content in controls (C), ectopic (EC), and eutopic (EU) endometrium of women with endometriosis. Results are expressed as mean ± S.E.M. of *n*: C = 9 (mPR*α*, mPR*β*, and mPR*δ*); EU = 9 (mPR*α*), 6 (mPR*β*), and 8 (mPR*δ*); and EC = 9 (mPR*α* and mPR*β*) and 7 (mPR*δ*). ∗*P* < 0.05 vs C.

mPR protein content was quantified by Western blot. mPR*α*, mPR*β*, and mPR*δ* content did not significantly change in the eutopic endometrium; however, in the ectopic endometrium of patients with endometriosis, the content of mPR*α* and mPR*β* was significantly lower than that in the endometrium of healthy women (Figure 2).

## 4. Discussion

Endometriosis is a chronic and inflammatory disease in which specific etiology has not been elucidated. However, some molecular and biochemical alterations have been related to the development and progression of this pathology [36]. Progesterone resistance is one of the classical hallmarks of endometriosis, and although the mechanisms involved in this resistance have not been fully explained, it has been suggested that a decrease in the expression of PR-B in the endometriotic lesions could be involved in this resistance [11]. PR is not the only receptor through which progesterone can exert its functions. mPRs belong to a group of cell surface receptors that activate nongenomic mechanisms of progesterone action in many progesterone-responsive cells and tissues [19]. In the present study, we have shown for the first time that gene expression and protein content of mPRs are decreased in the ectopic and eutopic endometrium of women with endometriosis compared to the endometrium of women without the disease, which in turn could explain another possibility for the molecular mechanisms involved in the lack of progesterone effects in this pathology.

The decrease in PR-B expression in the ectopic endometrium of patients with endometriosis only partially explains the progesterone resistance, since other factors such as alterations in progesterone signaling have been involved in this pathology [10, 11]. The results of the present study showed that both the expression of *PAQR7* and *PAQR5*, as well as the protein content of mPR*α* and mPR*β*, was significantly reduced in the ectopic endometrium of patients with endometriosis compared to the endometrium of women without the disease. Further studies are required to clarify whether the differences between mRNA expression and protein content are due to the sample size used in the present study or to specific mechanisms of transcriptional or translational regulation. These findings, together with previous studies, suggest that the decrease in the content of mPR*α*, mPR*β*, and PR-B in the ectopic endometrium of women with endometriosis contributes to the progesterone resistance observed in this disease.

In the present study, we were unable to confirm whether there was a dependence or correlation between eutopic and ectopic tissues in regard to mPR expression, since in most cases it was not possible to obtain both samples from the same patient. The dependence between samples with respect to the expression of mPRs is an interesting topic that deserves further investigation.

The decrease in the expression of PR-B in the eutopic endometrium of women with endometriosis remains controversial since some studies have not found this reduction [12]. In the present study, we have shown that the expression of *PAQR7*, *PAQR8*, *PAQR5*, and *PAQR6* genes is downregulated in the eutopic endometrium of patients with endometriosis compared to that of controls, which in turn could be associated with the progesterone resistance that leads to a reduced implantation capacity and increased risk of pregnancy loss observed in these patients [6]. However, we did not find a decrease in the protein content of mPR*α*, mPR*β*, and mPR*δ*, which should be addressed in future studies with a larger sample size to compare our findings at the mRNA level. We were not able to detect mPR*γ* protein due to technical limitations. A decrease in the expression and protein content of other membrane progesterone receptors, PGRMC1 and PGRMC2, has also been reported in the eutopic endometrium of patients with endometriosis compared to women without the disease [37]. The consistent decrease in mRNA levels of genes encoding mPRs and PGRMCs in the eutopic endometrium of women with endometriosis suggests a probable role of plasma membrane progesterone receptors in the pathogenesis of the disease, which should be addressed in future functional studies since it has been proposed that endometriosis originated from eutopic endometrium cells [38].

It has been previously reported that genes encoding mPRs are differentially expressed during the menstrual cycle, which suggests that sex hormones regulate their expression. Particularly, *PAQR7* expression is higher in the secretory phase of the menstrual cycle than in the proliferative phase, whereas the expression of *PAQR5* and *PAQR9* is decreased in the secretory phase and *PAQR8* expression is not differentially expressed during the menstrual cycle [30]. Half of the samples were obtained during the proliferative phase of the menstrual cycle in the present study, and the expression of the four genes analyzed (*PAQR7*, *PAQR8*, *PAQR5*, and *PAQR6*) was decreased in the eutopic endometrium of patients with endometriosis compared to the endometrium of control women. Further studies are required to compare the expression of *PAQR* genes in patients with endometriosis during different phases of the menstrual cycle. An open question that remains is whether the reduced expression of *PARQ* genes observed in patients with endometriosis is in part responsible for the progesterone resistance or if the latter leads to the decreased expression of those genes.

It has been recently reported that the expression of *PAQR7* and the respective protein content are decreased in endometrial cancer compared to adjacent unaffected tissue [35]. In the present study, we also found a decrease in the mRNA expression and protein content of mPR*α* in the ectopic endometrium of women with endometriosis, suggesting a possible connection between the alterations in endometriosis and endometrial cancer, as previously proposed [39].

## 5. Conclusions

The overall results of the present study demonstrate for the first time that gene expression of *PAQR7* and *PAQR5* and protein content of mPR*α* and mPR*β* are decreased in ectopic endometrium compared to that of women without the disease and that gene expression of *PAQR7*, *PAQR8*, *PAQR5*, and *PAQR6* is decreased in eutopic endometrium. Our results reinforce the theory of progesterone resistance as part of the etiology of endometriosis. Further studies are required to elucidate the functional role of mPRs in normal, eutopic, and ectopic endometrium.

## Figures and Tables

**Table 1:** Primers used in the present study.

Gene	Forward (5′-3′)	Reverse (5′-3′)	Reference
*18S*	CGCGGTTCTATTTTGTTGGT	AGTCGGCATCGTTTATGGTC	[27]
*PAQR7*	AACTGTCAAGGGAGGTGCTG	ATTGCATCCAGGCCATAATC	[27]
*PAQR8*	AGGACACAGCAAACAGGACA	GGCAACACAGGCAGGAATAA	[27]
*PAQR5*	CAGCTGTTTCACGTGTGTGTGATCCTG	GGACAGAAGTATGGCTCCAGCTATCTGAG	[35]
*PAQR6*	CTTTCATCTGGCTCCGTTTC	CTGGCAAACTGGATTACCT	Present study

**Table 2:** Characteristics of the women included in the present study.

Demographic/clinical characteristics	Patients (17)	Controls (17)
Age (mean, ±SD)	34.8 (±7.4)	31.9 (±9.6)
Term pregnancy (*n*)	10	11
Spontaneous abortion (*n*)	8	5
Pelvic pain (*n*)	17	0
Severe endometriosis (*n*)	8	Not applicable
Other lesions (*n*)	14	Not applicable
History of surgery for endometriosis before the present study (*n*)	8	Not applicable
Women recruited during proliferative phase (*n*)	8	6
Women recruited during secretory phase (*n*)	1	1
Women recruited at an unknown phase (*n*)	8	10

## Data Availability

The data used to support the findings of this study are included within the article.
